# From Detection to Decision: A Single-Center Analysis of Preoperative Imaging, Surgical Indications and Postoperative Histopathological Findings in Adrenal Lesions

**DOI:** 10.3390/diagnostics16172807

**Published:** 2026-09-01

**Authors:** Feride Pınar Altay, Şefika Burçak Polat, Sevgül Fakı, Gülsüm Karaahmetli, Leyla Akdoğan, Oya Topaloğlu, Reyhan Ersoy, Bekir Çakır

**Affiliations:** 1 Department of Endocrinology and Metabolism, Ankara Bilkent City Hospital, Ankara 06800, Türkiye; black_snowtr@yahoo.com (S.F.); gulsumgedik85@gmail.com (G.K.); 2 Department of Endocrinology and Metabolism, Faculty of Medicine, Ankara Yıldırım Beyazıt University, Ankara 06010, Türkiye; burcakugurlu@gmail.com (Ş.B.P.); oyasude@gmail.com (O.T.); reyhanersoy@yahoo.com.tr (R.E.); drcakir@yahoo.com (B.Ç.); 3 Department of Endocrinology and Metabolism, Kırıkkale High Specialization Hospital, Kırıkkale 71450, Türkiye; leyla.khrmn@hotmail.com

**Keywords:** adrenal lesion, adrenal incidentaloma, adrenalectomy, benign, malignant

## Abstract

**Background/Objectives:** The aim of this retrospective study was to characterize the preoperative hormonal, biochemical, and imaging features of adrenal lesions (ALs) and to assess differences in these features between benign and malignant lesions. **Methods:** This was a retrospective, single-center observational study conducted at the Endocrinology Clinic of Ankara Bilkent City Hospital. We reviewed the medical records of all patients referred to our clinic for the evaluation of an AL between February 2019 and August 2025. Demographic and clinical data and initial imaging were extracted from electronic medical records. **Results:** A total of 157 patients who underwent adrenalectomy were included in the analysis. Of these, 98 patients were female (62.4%) and 59 were male (37.6%). The mean age of the patients was 51.2 ± 12.54 years. Functionally, the findings were pheochromocytoma (n:44), overt Cushing syndrome (n:17), mild autosomal cortisol secretion (n:29), primary hyperaldosteronism (n:18), and non-functional (n:47). One patient had pheochromocytoma and mild autosomal cortisol secretion, and one patient had overt Cushing syndrome and primary hyperaldosteronism. Of the adrenal lesions, 70 (44.6%) were on the right, 71 (45.2%) on the left, and 16 (10.2%) were bilateral. 9% (7/79) of lesions measuring 4 cm and above, and 14% (4/28) of lesions measuring 6 cm and above were found to be malignant. The malignancy rate was 22% (4/18) in lesions located on the right and measuring 6 cm and above. All adrenocortical carcinomas were larger than 4 cm (8.3 ± 3.46) and all were on the right. The highest-grade malignant epithelial tumor was 12.9 cm and on the right. The malignant pheochromocytoma was 5.2 cm and on the left. It was determined that the frequency of right adrenal localization was significantly higher in the malignant group (85.7%) compared to the benign group (42.7%) (*p* = 0.045). Right tumor diameter (*p* = 0.002) and right and/or left tumor diameter (*p* < 0.001) were significantly higher in malignant lesions compared to benign lesions. Logistic regression analysis also supported a significant relationship between tumor size and malignancy. **Conclusions:** In our study, 9% (7/79) of lesions measuring 4 cm and above, and 14% (4/28) of lesions measuring 6 cm and above were found to be malignant. The malignancy rate in lesions on the right side measuring 6 cm and above was determined to be 22% (4/18). Although benign masses constituted the majority of lesions measuring 4 cm and above, all adrenocortical carcinomas were above 4 cm (8.3 ± 3.46). In light of the current findings, tumor size was observed to be associated with the distinction between malignant and benign adrenal lesions and may be considered an important descriptive characteristic in the clinical assessment of adrenal lesions.

## 1. Introduction

Advances in imaging technology have led to a growing number of incidentally detected adrenal masses, making adrenal incidentalomas (AIs) a common clinical finding [1,2]. An AI is an adrenal lesion ≥ 1 cm discovered during imaging performed for reasons unrelated to adrenal disease [3]. The prevalence of AIs increases with age, reaching approximately 7% in older adults. AI is a common endocrine diagnosis affecting about 2% of the general population, but over 7% of those over 70 years [3,4]. While most lesions are benign and non-functioning adenomas, some may be hormonally active or malignant. Therefore, the evaluation of an AL focuses on two key questions: whether the lesion is benign or malignant and whether it produces excess adrenal hormones. Accurate assessment requires a combination of imaging findings, biochemical testing, and clinical evaluation. This integrated approach helps identify clinically significant lesions while minimizing unnecessary investigations and treatments.

The systematic evaluation of any newly identified AL revolves around two fundamental and interrelated clinical questions: whether the lesion exhibits biochemical evidence of hormonal excess and whether its morphological and radiological characteristics suggest a benign or malignant etiology [3]. Addressing these questions demands a structured, multimodal approach that integrates high-resolution imaging characteristics, comprehensive biochemical profiling, and thorough clinical assessment. Several leading international endocrine societies, including the European Society of Endocrinology in collaboration with the European Network for the Study of Adrenal Tumors (ESE/ENSAT), the Korean Endocrine Society (KES), and the American Association of Clinical Endocrinologists together with the American Association of Endocrine Surgeons (AACE/AAES), have developed evidence-based guidelines to standardize this evaluation process [3,5,6,7]. Nevertheless, despite the availability of these well-established frameworks, real-world adherence to guideline recommendations remains incompletely characterized, and the gap between recommended practice and actual clinical behavior represents an important and underexplored area of investigation.

Among 792 patients evaluated for ALs at our Endocrinology Clinic between 2019 and 2025, 157 underwent adrenalectomy and had definitive histopathological diagnoses. This large single-center cohort provided a unique opportunity to investigate the clinical factors associated with malignant adrenal lesions. The primary aim of this retrospective study was to characterize the preoperative hormonal, biochemical, and imaging features of adrenal incidentalomas (ALs) and to assess differences in these features between benign and malignant lesions.

## 2. Materials and Methods

### 2.1. Study Design and Patient Population

This was a retrospective, single-center observational study conducted at the Endocrinology Clinic of Ankara Bilkent City Hospital. We reviewed the medical records of all patients referred to our clinic for the evaluation of an AI between February 2019 and August 2025. A total of 792 patients met the inclusion criteria and formed the study population. Of these, 157 patients underwent adrenalectomy and had histopathologically confirmed diagnoses, constituting the surgical subgroup used for the primary malignancy prediction analysis.

Patients were excluded if the adrenal mass was identified in the context of a known extra-adrenal malignancy with suspected metastatic disease, or if imaging had been performed explicitly to evaluate adrenal pathology. Patients with incomplete medical records or those lost to follow-up before completion of the initial diagnostic workup were also excluded.

### 2.2. Data Collection

Demographic and clinical data and initial imaging were extracted from electronic medical records. All data were collected by trained investigators using a standardized data extraction form to ensure consistency.

### 2.3. Imaging Assessment

In patients who did not undergo adrenal gland imaging, computed tomography (CT) without contrast was performed as part of the initial evaluation. Recorded imaging parameters included lesion size (maximum diameter in centimeters), laterality of the lesion, and morphological characteristics. CT or magnetic resonance imaging (MRI) findings with contrast were also recorded when available. Alternative imaging methods were used when necessary.

### 2.4. Hormonal Evaluation

Hormonal evaluation was carried out in accordance with the guidance issued by the ESE/ENSAT, KES, and AACE/AAES, and the specific tests performed at our institution were documented and analyzed accordingly. Autonomous cortisol secretion was assessed in all patients using the 1 mg overnight dexamethasone suppression test, with a post-DST serum cortisol level exceeding 50 nmol/L (1.8 µg/dL) in the absence of overt clinical features of Cushing syndrome taken as indicative of mild autonomous cortisol secretion (MACS). Pheochromocytoma was excluded through measurement of 24 h urinary fractionated metanephrines and/or plasma free metanephrines, with values surpassing the upper limit of the local laboratory reference range prompting further clinical evaluation. Primary aldosteronism was screened using the aldosterone-to-renin ratio in all patients with hypertension or unexplained hypokalemia, and confirmatory testing was pursued where the clinical picture warranted it. In cases where the lesion’s imaging characteristics or the patient’s clinical presentation raised concern for adrenocortical carcinoma or a sex-hormone-secreting tumor, serum DHEA-S (dehydroepiandrosterone-sulfate), testosterone, and estradiol levels were additionally obtained. Finally, to evaluate real-world adherence to guideline recommendations, the hormonal tests performed for each patient were systematically compared against those mandated by the applicable guidelines based on that individual’s clinical and radiological profile.

### 2.5. Histopathological Evaluation

In the 157 patients who underwent adrenalectomy, histopathological diagnoses were established by experienced pathologists according to the World Health Organization (WHO) classification of endocrine tumors [8]. Lesions were classified as benign (adrenocortical adenoma, myelolipoma, cyst, ganglioneuroma, or other benign entities), adrenocortical carcinoma (ACC), pheochromocytoma, metastatic carcinoma, or other malignant tumors. The Weiss scoring system was applied for the assessment of adrenocortical neoplasms, and a score ≥3 was used to define malignancy [9].

### 2.6. Statistics

Continuous variables were expressed as mean ± standard deviation (SD) or median with interquartile range (IQR), as appropriate, based on the results of normality testing using the Shapiro–Wilk test. Categorical variables were summarized as number (n) and percentage (%). The Chi-square test was used to compare the demographic, clinical, biochemical, and imaging characteristics of benign and malignant adrenal lesions for categorical variables. Fisher’s Exact Test was applied to 2 × 2 tables and the Fisher–Freeman–Halton Exact Test was applied to larger (rxc) tables where the assumptions of the Chi-square test could not be met. The Mann–Whitney U test was used to compare continuous variables between two groups. To evaluate variables associated with malignancy, univariate binary logistic regression analysis was applied separately for each variable. Logistic regression results were reported using regression coefficient (B), standard error (SE), Wald statistic, odds ratio (OR), 95% confidence interval (95% CI), and *p*-values. Model fit was assessed using the Hosmer–Lemeshow goodness-of-fit test where appropriate. A significance level of *p* < 0.05 was accepted for all statistical analyses. Study data were imported and analyzed using IBM SPSS Statistics 26 (IBM Corp., Armonk, NY, USA).

## 3. Results

**Figure 1:**
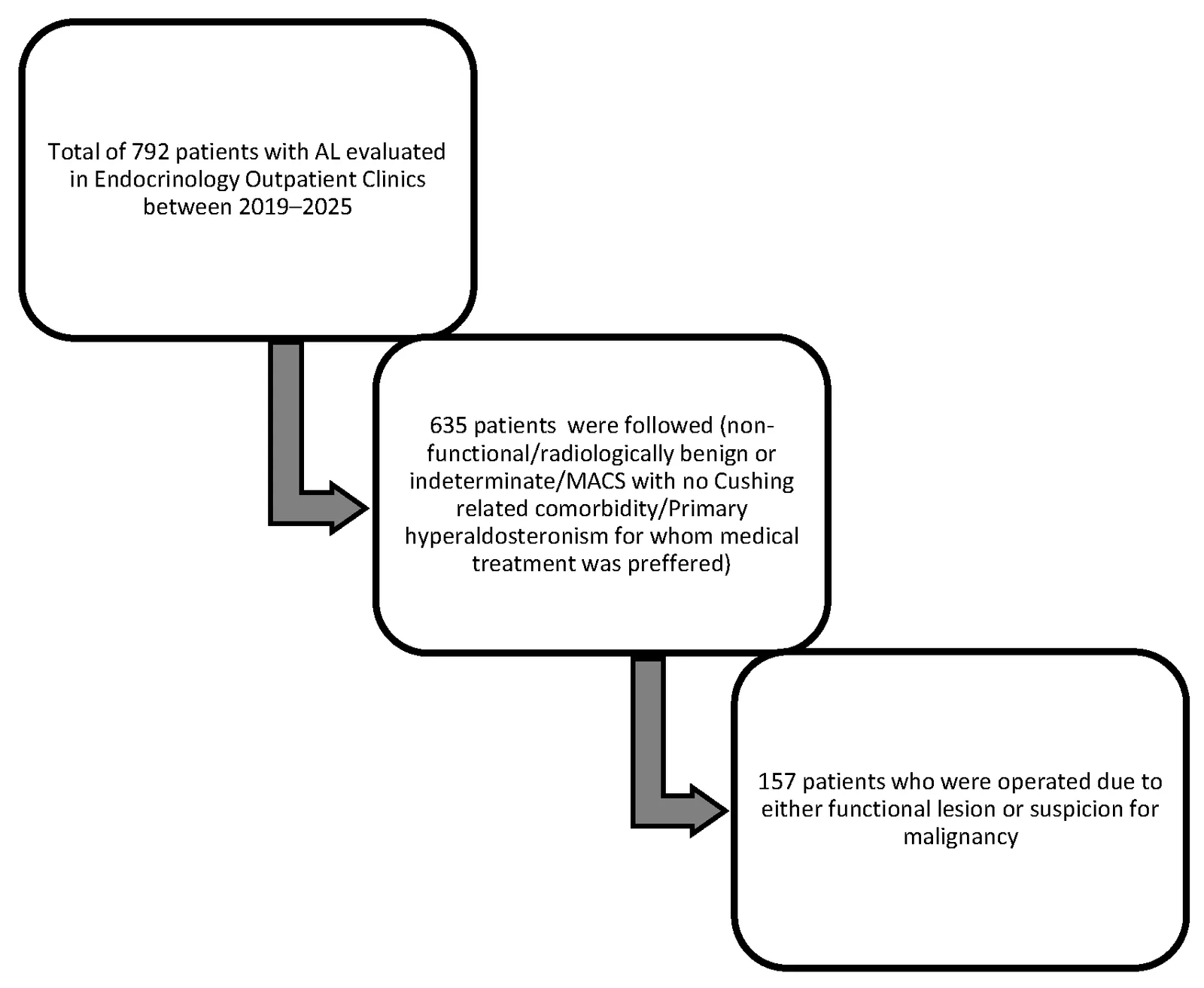
Selection flowchart of AL patients. AL: Adrenal lesion, MACS: Mild autonomous cortisol secretion.

A total of 157 patients who underwent adrenalectomy were included in the analysis. Figure 1 indicates the selection flowchart of AL patients.

The mean age of the cohort was 51.2 ± 12.54 years, with a predominance of female patients (*n* = 98, 62.4%) over male patients (*n* = 59, 37.6%). Regarding tumor laterality, the distribution was nearly equal between the right (*n* = 70, 44.6%) and left (*n* = 71, 45.2%) adrenal glands, while bilateral lesions were identified in 16 patients (10.2%). The mean tumor size was 4.37 ± 2.49 cm on the right side and 3.57 ± 2.32 cm on the left side. Table 1 indicates the demographic and clinical characteristics of patients.

**Figure 2:**
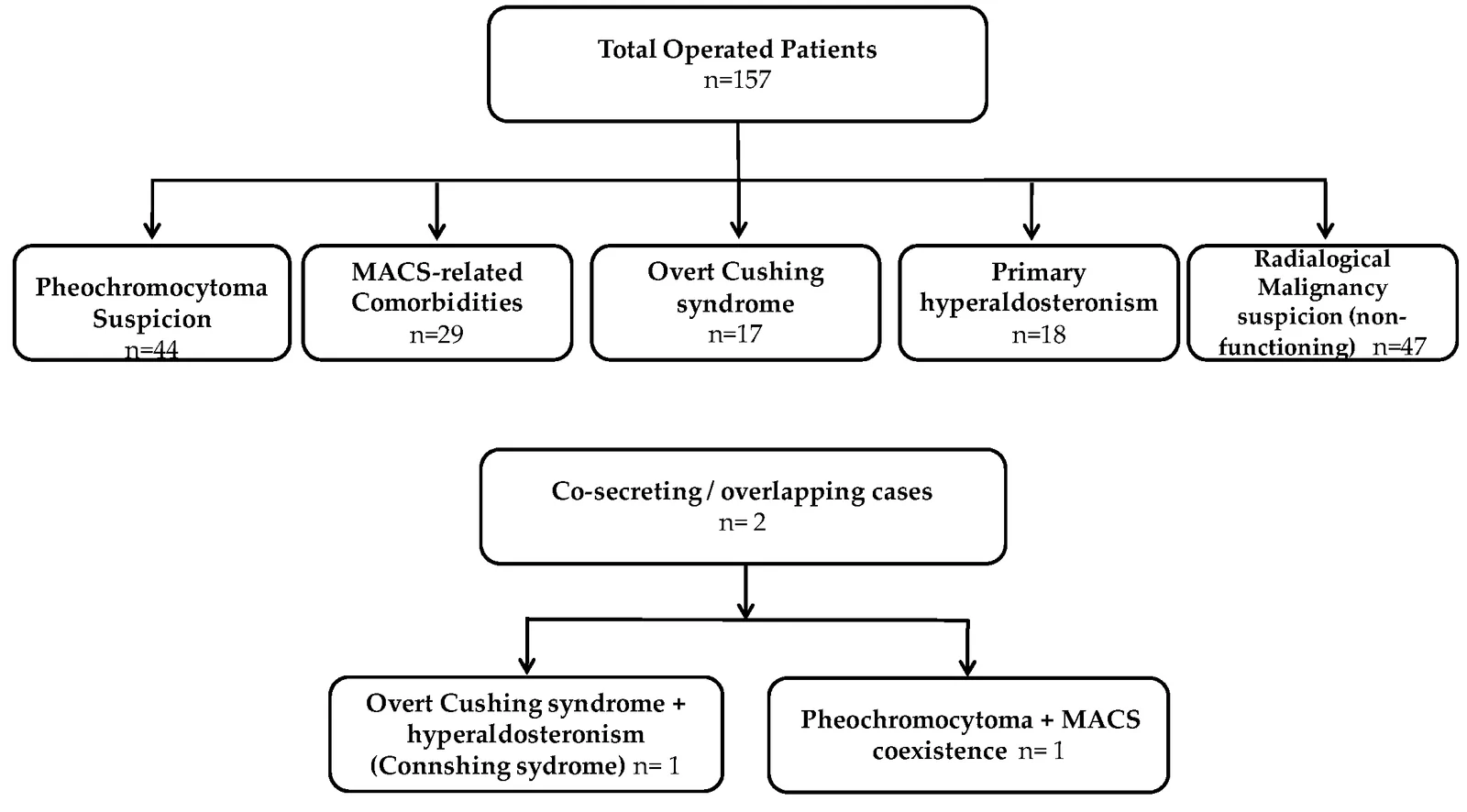
Indications for adrenalectomy according to functional status. MACS: Mild autonomous cortisol secretion.

With respect to endocrine function, the largest subgroup consisted of patients with radiologically suspicious lesions operated on for concern of malignancy, who were found to be non-functioning on hormonal workup (*n* = 47). Pheochromocytoma accounted for the second most common indication (*n* = 44), followed by MACS associated with uncontrolled comorbidities such as hypertension, diabetes mellitus, or osteoporosis (*n* = 29), primary hyperaldosteronism (*n* = 18), and overt Cushing syndrome (*n* = 17). Two patients presented with co-secreting tumors: one exhibited features of both overt Cushing syndrome and primary hyperaldosteronism, consistent with Connshing syndrome, and one demonstrated coexistence of pheochromocytoma and mild autonomous cortisol secretion. Figure 2 shows the distribution of operated AL patients according to endocrine function.

**Figure 3:**
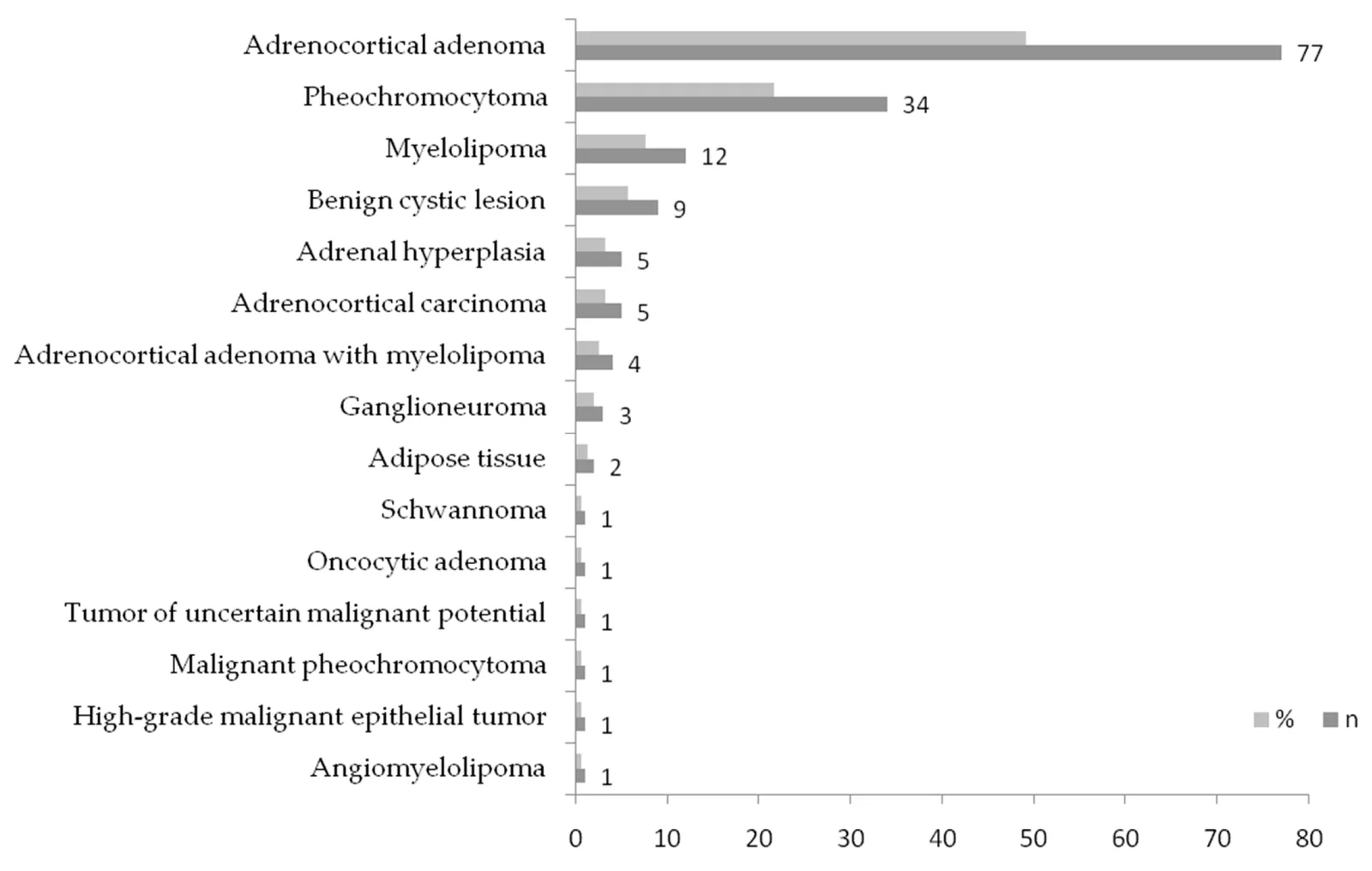
Histopathological diagnosis of patients.

The majority were benign, with adrenocortical adenoma being the most common diagnosis at 77 cases (49%), followed by pheochromocytoma in 34 cases (21.7%) and myelolipoma in 12 cases (7.6%). Benign cystic lesions were identified in nine patients, while adrenal hyperplasia and adrenocortical carcinoma each accounted for five cases. Four patients had a combined finding of adrenocortical adenoma with myelolipoma. Ganglioneuroma was present in three cases and isolated adipose tissue in two. The remaining seven cases fell under miscellaneous categories, each occurring once: angiomyelolipoma, high-grade malignant epithelial tumor, malignant pheochromocytoma, tumor of uncertain malignant potential, oncocytic adenoma, and schwannoma. Overall, the vast majority of lesions were benign, with malignant or potentially malignant tumors representing a small minority of the series. Figure 3 shows the histopathological diagnosis of patients.

Among 157 patients with adrenal lesions, 70 (44.6%) were right-sided, 71 (45.1%) were left-sided, and 16 (10.2%) were bilateral. Seven patients (4.5%) were diagnosed with malignant adrenal lesions. The malignancy rate was 9% (7/79) among lesions measuring 4 cm or greater and increased to 14% (4/28) among lesions measuring 6 cm or greater, rising further to 22% (4/18) in right-sided lesions measuring 6 cm or above. All adrenocortical carcinomas exceeded 4 cm in diameter (mean 8.3 ± 3.46 cm) and were exclusively right-sided. The high-grade malignant epithelial tumor measured 12.9 cm and was right-sided, whereas the malignant pheochromocytoma measured 5.2 cm and was left-sided.

Table 2 and Table 3 compare the demographic, clinical, and imaging characteristics of benign and malignant adrenal lesions. It was determined that the frequency of right adrenal localization was significantly higher in the malignant group (85.7%) compared to the benign group (42.7%) (*p* = 0.045) (Table 3).

Right tumor diameter (*p* = 0.002), right and/or left tumor diameter (*p* < 0.001), and SUV value (*p* = 0.019) were significantly higher in malignant lesions compared to benign lesions. Furthermore, PET imaging was performed in the vast majority of malignant cases (*p* < 0.001) (Table 3).

However, no statistically significant differences were found between benign and malignant groups in terms of gender, bilateral involvement, left adrenal location, endocrine functional status, cystic or necrotic appearance on imaging, signal loss, diffusion restriction, heterogeneous structure, age, and DHEAS level (*p* > 0.05).

Univariate binary logistic regression analysis was applied to evaluate the relationship between right tumor diameter and malignancy. The established model was found to be statistically significant (χ^2^ = 16.629; *p* < 0.001). The Hosmer–Lemeshow goodness-of-fit test showed that the model was consistent with the data (χ^2^ = 14.234; *p* = 0.076). Accordingly, each 1 cm increase in tumor diameter increases the probability of malignancy by approximately 1.813 times. As a result of the analysis, it was determined that tumor diameter was significantly associated with malignancy (OR = 1.813; 95% CI = 1.296–2.537; *p* < 0.001) (Table 4).

Univariate binary logistic regression analysis was applied to evaluate the relationship between tumor size right and/or left diameter and malignancy. The established model was found to be statistically significant (χ^2^ = 16.699; *p* < 0.001). The Hosmer–Lemeshow goodness-of-fit test showed that the model was consistent with the data (χ^2^ = 9.933; *p* = 0.270). Accordingly, each 1 cm increase in tumor diameter increases the probability of malignancy by approximately 1.670 times. As a result of the analysis, it was determined that tumor diameter was significantly associated with malignancy (OR = 1.670; 95% CI = 1.283–2.174; *p* < 0.001) (Table 5).

We performed 18 F-FDG (Fluorodeoxyglucose F 18) PET/CT in 34 of the total cohort (21.7%). Of these, 29 (85.3%) demonstrated increased tracer uptake (PET-positive), while five (14.7%) showed no significant uptake (PET-negative). Cross-referencing with final histopathological diagnosis, all six malignant lesions in the PET-imaged subgroup were correctly identified as PET-positive, yielding a sensitivity of 100%. However, 23 of the 28 benign lesions also demonstrated uptake, resulting in a low specificity of 17.9%, a positive predictive value (PPV) of 20.7%, and a negative predictive value (NPV) of 100%, with 95% CI for both values (Table 6).

Univariate binary logistic regression analysis was applied to evaluate the relationship between SUV value and malignancy. The established model was found to be statistically significant (χ^2^ = 10.057; *p* = 0.002). The Hosmer–Lemeshow goodness-of-fit test revealed that the model fit the data well (χ^2^ = 9.851; *p* = 0.276). Accordingly, each 1-unit increase in SUV value increases the probability of malignancy by approximately 1.382 times. As a result of the analysis, it was determined that SUV value is significantly associated with malignancy (OR = 1.382; 95% CI = 1.095–1.743; *p* = 0.006) (Table 7).

Univariate binary logistic regression analysis was applied to evaluate the association between PET imaging and malignancy. The established model was found to be statistically significant (χ^2^ = 13.924; *p* < 0.001). The analysis revealed that the probability of malignancy was 26.143 times higher in patients who underwent PET imaging compared to those who did not (OR = 26.143; 95% CI = 3.026–225.890; *p* = 0.003).

A total of 22 patients underwent GA-68 DOTATATE PET imaging for the evaluation of pheochromocytoma. Of these, 18 patients demonstrated radiotracer uptake on PET (scan-positive), while four showed no uptake (scan-negative). Among the 12 patients with confirmed pheochromocytoma on the reference standard, GA-68 DOTATATE PET correctly identified 11 (true positives) and missed one (false negative). Among the 10 patients without pheochromocytoma, three were correctly identified as scan-negative (true negatives), while seven yielded false-positive results. GA-68 DOTATATE PET demonstrated a sensitivity of 91.7% (11/12) and a specificity of 30.0% (3/10) for the detection of pheochromocytoma. The PPV was 61.1% (11/18) and the NPV was 75.0% (3/4), with an overall accuracy of 63.6% (14/22) (Table 8).

## 4. Discussion

The present series of 157 adrenalectomies performed among 792 patients evaluated for adrenal lesions at our institution yields a surgical referral rate of 19.8%, which falls within the range reported by contemporary single-center cohorts and is broadly consistent with published estimates. The present series of 157 adrenalectomies performed among 792 patients evaluated for adrenal lesions at our institution yields a surgical referral rate of 19.8%, which falls within the range reported by contemporary single-center cohorts. Previous studies have reported adrenalectomy rates of approximately 20–23% among patients evaluated for adrenal incidentalomas, supporting the representativeness of our surgical practice and referral patterns [10,11]. The female predominance observed in our cohort (62.4%) is consistent with previously reported series and is largely attributable to the higher prevalence of adrenocortical adenomas and functioning tumors such as primary aldosteronism and cortisol-secreting lesions in women [12,13]. Most adrenal incidentalomas are adrenocortical adenomas that do not cause harm and do not require surgery, but a non-negligible proportion is represented by functionally active masses [3], including cortisol-secreting adenomas, pheochromocytomas, aldosterone-secreting adenomas, and malignant nodules such as adrenocortical carcinomas, all of which may require operative intervention. In tertiary referral centers, however, surgical rates are inherently higher due to case selection. A recent retrospective cohort study from a tertiary urology center reported that 87.5% of referred patients ultimately underwent adrenalectomy, with functional tumors comprising 54% of the surgical cohort [14]. The surgical rate of 19.8% in our series therefore reflects appropriate case selection within a referral center context, where imaging-indeterminate lesions, functioning tumors, and malignancy-suspicious masses are concentrated.

The most common histological diagnosis is adrenocortical adenoma. The fact that adrenal adenoma is the most common type is generally consistent with surgical series in the literature. The indications for adrenalectomy encompass functional adrenal tumors regardless of size, suspicion or presence of malignancy, and non-functional tumors with malignancy risk, typically those larger than 4 cm in diameter. The largest functional subgroup in our series consisted of patients operated on for radiological suspicion of malignancy with non-functioning tumors on hormonal workup (*n* = 47), reflecting the evolving clinical emphasis on imaging-based risk stratification. The revised 2023 European Society of Endocrinology (ESE)/ENS@T guideline defines indeterminate adrenal masses as homogeneous lesions with 11–20 HU and size ≥ 4 cm, homogeneous lesions with >20 HU and size < 4 cm, and heterogeneous tumors < 4 cm, each warranting further functional imaging workup [3].

The co-secreting tumors identified in our series one with concurrent overt Cushing syndrome and primary hyperaldosteronism (so-called Connshing syndrome) and one with coexisting pheochromocytoma and mild autonomous cortisol secretion (MACS) represent rare but increasingly recognized entities. Current ESE guidelines recommend that patients with adrenal incidentalomas be discussed in a multidisciplinary expert team meeting when imaging is not consistent with a benign lesion, when evidence of hormone excess exists, when adrenal surgery is considered, or when significant tumor growth is detected on follow-up imaging [3].

Regarding tumor size and malignancy risk, the overall malignancy rate in our cohort was 4.5% (7/157). Right tumor diameter (*p* = 0.002) and right and/or left tumor diameter (*p* < 0.001) were significantly higher in malignant lesions compared to benign lesions. Logistic regression analysis also supported a significant relationship between tumor size and malignancy. It was determined that 9% of lesions measuring 4 cm and above, and 14% of lesions measuring 6 cm and above, were malignant. This finding aligns with the established principle that larger lesion size carries a substantially higher risk of malignancy, and supports the guideline recommendation that size ≥4 cm should prompt surgical consideration, particularly in the absence of definitive benign imaging characteristics. The 2023 ESE guideline clarifies that a homogeneous adrenal mass with ≤10 Hounsfield units on non-contrast CT requires no further follow-up irrespective of size, and introduces steroid metabolomics using tandem mass spectrometry as an adjunct tool to discriminate malignancy. The exclusive right-sided occurrence and large mean diameter (8.3 ± 3.46 cm) of adrenocortical carcinomas in our series. It was determined that the frequency of right adrenal localization was significantly higher in the malignant group (85.7%) compared to the benign group (42.7%) (*p* = 0.045). Adrenocortical carcinoma is a rare, aggressive malignancy with an annual incidence of approximately 1 case per million population, and differentiating ACC from benign adrenocortical tumors remains challenging due to the limited specificity of standard diagnostic imaging. Population-based data suggest that tumor size is a crucial prognostic factor in ACC, with an optimal cutoff of approximately 8.5 cm associated with significantly worse cancer-specific and overall survival [15].

Implementation of the 2016 guidelines led to measurable improvements in clinical practice, including a reduction in unnecessary surgical interventions and an increased rate of discharge for patients with benign adrenal lesions, ultimately optimizing healthcare resource utilization and reducing patient burden [16].

The study by Pantalone K.M. et al., the largest using surgical histopathology as the gold standard for diagnosis, identified change in adrenal mass size as a significant predictor of malignancy [17]. A 4 cm cutoff provides the best balance of sensitivity and specificity, maintaining specificity comparable to higher thresholds while showing a tendency toward improved sensitivity [18]. Tumor size, tumor weight, hormonal activity, and the Weiss system are valuable clinicopathological parameters that enable accurate differentiation in most cases of adrenocortical carcinoma and adenoma [19].

With respect to functional imaging, ^18^F-FDG PET/CT was performed in 34 patients (21.7%) and demonstrated a sensitivity of 100% for malignancy detection, with all six malignant lesions in this subgroup correctly identified. However, specificity was markedly low at 17.9%, with 23 of 28 benign lesions also demonstrating tracer uptake. This low specificity is a well-recognized limitation of FDG PET in the adrenal context, largely attributable to false-positive uptake in pheochromocytomas and other metabolically active benign lesions. A landmark meta-analysis by Boland et al. encompassing 1391 lesions reported a mean sensitivity of 97% and specificity of 91% for FDG PET in differentiating malignant from benign adrenal disease; the substantially lower specificity in our series likely reflects the high proportion of pheochromocytomas in the imaged subgroup, as these tumors are known to exhibit avid FDG uptake irrespective of their benign biological behavior [20]. A prospective multicentric study identified a tumor-to-liver SUVmax ratio greater than 1.5 as the optimal cutoff for predicting malignancy, with sensitivity of 86.7%, specificity of 86.1%, PPV of 56.5%, and NPV of 96.9% [21]. SUV values (*p* = 0.019) were significantly higher in malignant lesions compared to benign lesions. Furthermore, PET imaging was performed in the vast majority of malignant cases. As a result of the logistic regression analysis, it was determined that SUV value is significantly associated with malignancy (OR = 1.382; 95% CI = 1.095–1.743; *p* = 0.006). Accordingly, each 1-unit increase in SUV value increases the probability of malignancy by approximately 1.382 times. The observed association between PET performance and malignancy should be interpreted cautiously, as PET was performed selectively based on clinical indications. Therefore, this association may largely reflect selection or indication bias rather than an independent predictive value of PET for malignancy.

For the evaluation of pheochromocytoma, ^68^Ga-DOTATATE PET was performed in 22 patients and demonstrated a sensitivity of 91.7% and NPV of 75% for confirmed pheochromocytoma, consistent with its established role as a highly sensitive somatostatin receptor-based imaging modality. The specificity was, however, low at 30%, with 7 of 10 non-pheochromocytoma patients yielding false-positive results. Comparative studies have shown that ^68^Ga-DOTATATE PET/CT detects a similar number of lesions to ^18^F-FDG PET/CT but with significantly greater lesion-to-background contrast, and given its high specificity, patient convenience, and relatively low cost, DOTATATE PET/CT has been proposed as the ideal first-line functional investigation for pheochromocytoma and paraganglioma. The reduced specificity in our cohort may reflect the inclusion of other somatostatin receptor-expressing adrenal lesions such as adrenocortical neoplasms or neuroendocrine tumors within the imaged population. It is increasingly recognized that the sensitivity and specificity of ^68^Ga-DOTATATE vary substantially depending on the biological heterogeneity of pheochromocytomas, with most published studies focusing on metastatic lesion identification rather than primary tumor diagnosis, and limited knowledge existing regarding diagnostic limitations for localized primary pheochromocytoma.

Several limitations of the present study warrant acknowledgment. The retrospective single-center design introduces potential selection bias, and the relatively small number of malignant cases (*n* = 7) limits the statistical power of size-based subgroup analyses. The absence of SUV-based quantitative thresholds for FDG PET interpretation and the lack of genetic profiling data for pheochromocytoma patients preclude more granular functional imaging comparisons. Future prospective multicenter studies incorporating steroid metabolomics, standardized PET quantification, and germline mutation analysis would strengthen the evidence base for individualized risk stratification in adrenal surgical series.

## 5. Conclusions

In our study, 9% of lesions measuring 4 cm and above, and 14% of lesions measuring 6 cm and above were found to be malignant. The malignancy rate in lesions on the right side measuring 6 cm and above was determined to be 22%. Although benign masses constituted the majority of lesions measuring 4 cm and above, all adrenocortical carcinomas were above 4 cm (8.3 ± 3.46). Right tumor diameter (*p* = 0.002) and right and/or left tumor diameter (*p* < 0.001) were significantly higher in malignant lesions compared to benign lesions. Logistic regression analysis also supported a significant relationship between tumor size and malignancy. Overall, malignant adrenal lesions tended to be larger than benign lesions, indicating that tumor size remains a relevant clinical characteristic when describing and evaluating adrenal lesions.

## Figures and Tables

**Table 1:** Demographic and clinical characteristics of patients.

Variable	Value
Age (years)	51.2 ± 12.54
Sex (*n*)	
Female	98 (62.4%)
Male	59 (37.6%)
Tumor laterality (*n*)	
Right	70 (44.6%)
Left	71 (45.2%)
Bilateral	16 (10.2%)
Tumor size (cm)	
Right	4.37 ± 2.49
Left	3.57 ± 2.32

**Table 2:** Comparison of demographic, clinical, biochemical, and imaging characteristics of benign and malignant adrenal lesions.

	Benign (*n* = 150)	Malign (*n* = 7)	χ ^2^	*p*
Sex				
Female	96 (64.0)	2 (28.6)	3.579 _c_	0.104
Male	54 (36.0)	5 (71.4)		
Bilateral				
No	234 (89.3)	7 (100.0)	0.831 _c_	1.000
Yes	16 (10.7)	0 (0.0)		
Right				
No	86 _a_ (57.3)	1 _b_ (14.3)	5.016 _c_	0.045 *
Yes	64 _a_ (42.7)	6 _b_ (85.7)		
Left				
No	80 (53.3)	6 (85.7)	2.831 _c_	0.129
Yes	70 (46.7)	1 (14.3)		
Endocrine function				
Non-functioning	42 (28.0)	5 (71.4)	4.155 _d_	0.275
Cushing syndrome	18 (12.0)	0 (0.0)		
Pheochromocytoma	44 (29.4)	1 (14.3)		
Mild autonomous cortisol secretion	28 (18.7)	1 (14.3)		
Primary hyperaldosteronism	18 (12.0)	0 (0.0)		

* *p* < 0.05, χ^2^: Pearson Chi-square test; c: Fisher’s Exact Test; d: Fisher–Freeman–Halton Exact Test. a,b: Statistically significant difference between ratios with different superscript letters in the same row (*p* < 0.05).

**Table 3:** Comparison of demographic, clinical, biochemical, and imaging characteristics of benign and malignant adrenal lesions.

	Benign (*n* = 150)	Malign (*n* = 7)	Z	*p*
	Median (Min-Max)	Median (Min-Max)		
Age (years)	52 (19–79)	55 (36–73)	−0.851	0.395
DHEA-S (µg/dL)	75.5 (3.3–663.3)	146 (21.7–1937.0)	−1.131	0.258
Tumor Size Right (cm)	3.6 (0.9–12.0)	10.5 (4.5–12.9)	−3.127	0.002 **
Tumor Size Right and/or Left (cm)	3.6 (0.9–13.0)	10.0 (4.5–12.9)	−3.334	<0.001
SUV	3.3 (0–15.3)	13.1 (2.0–15.2)	−2.353	0.019 *

* *p* < 0.05, ** *p* < 0.01, Z: Mann–Whitney U Test.

**Table 4:** Results of univariate logistic regression analysis evaluating the relationship between right tumor diameter and malignancy.

Independent Variable	B	SE	Wald	sd	*p*	Exp (B) (OR)	95% CI for OR	
							Bottom	Top
Tumor Size Right (cm)	0.595	0.171	12.070	1	<0.001	1.813	1.296	2.537
Constant	−6.329	1.410	20.148	1	<0.001	0.002		
Model Summary: χ^2^ = 16.629 *p* ≤ 0.001 CCR = 95.6% Hosmer and Lemeshow Test; χ^2^ = 14.234 *p* = 0.076								

B: Regression coefficient; Exp (B): Odds ratio (OR); SE: standard error; CI: Confidence interval; CCR: Correct Classification Rate; *p* < 0.05 is considered statistically significant.

**Table 5:** Results of univariate logistic regression analysis evaluating the relationship between right and/or left tumor diameter and malignancy.

Independent Variable	B	SE	Wald	sd	*p*	Exp (B) (OR)	95% CI for OR	
							Bottom	Top
Tumor Size Right and/or Left (cm)	0.513	0.134	14.539	1	<0.001	1.670	1.283	2.174
Constant	−6.049	1.092	30.706	1	<0.001	0.002		
Model Summary: χ^2^ = 16.699 *p* ≤ 0.001 CCR = 94.9% Hosmer and Lemeshow Test; χ^2^ = 9.933 *p* = 0.270								

B: Regression coefficient; Exp (B): Odds ratio (OR); SE: standard error; CI: Confidence interval; CCR: Correct Classification Rate; *p* < 0.05 is considered statistically significant.

**Table 6:** Patient numbers including PET (positron emission tomography) uptake in malignant and benign groups.

	Malignant (*n*)	Benign (*n*)	Total (*n*)
PET Positive	6	23	29
PET Negative	0	5	5
Total	6	28	34

**Table 7:** Results of univariate logistic regression analysis evaluating the relationship between SUV value and malignancy.

Independent Variable	B	SE	Wald	sd	*p*	Exp (B) (OR)	95% CI for OR	
							Bottom	Top
SUV	0.323	0.118	7.466	1	0.006	1.382	1.095	1.743
Constant	−3.856	1.149	11.268	1	<0.001	0.021		
Model Summary: χ^2^ = 10.057 *p* = 0.002 CCR = 91.2% Hosmer and Lemeshow Test; χ^2^ = 9.851 *p* = 0.276								

B: Regression coefficient; Exp (B): Odds ratio (OR); SE: standard error; CI: Confidence interval; CCR: Correct Classification Rate; *p* < 0.05 is considered statistically significant.

**Table 8:** Number of patients with GA-68 DOTATATE uptake in pheochromocytoma.

	GA-68 DOTATATE Positive (*n*)	GA-68 DOTATATE Negative (*n*)	Total (*n*)
PHEO Positive	11 (TP)	1 (FN)	12
PHEO Negative	7 (FP)	3 (TN)	10
Total	18	4	22

PHEO: Pheochromocytoma.

## Data Availability

The original contributions presented in this study are included in the article. Further inquiries can be directed to the corresponding author.

## Abbreviations

The following abbreviations are used in this manuscript:

AACE	American Association of Clinical Endocrinologists
AAES	American Association of Endocrine Surgeons
ACC	Adrenocortical carcinoma
AI	Adrenal incidentalomas
AL	Adrenal lesion
CT	Computed tomography
DHEA-S	Dehydroepiandrosterone sulfate
ENSAT	European Network for the Study of Adrenal Tumors
ESE	European Society of Endocrinology
FDG	Fluorodeoxyglucose
HU	Hounsfield units
IQR	Interquartile range
KES	Korean Endocrine Society
MACS	Mild autonomous cortisol secretion
MRI	Magnetic resonance imaging
NPV	Negative predictive value
OR	Odds ratios
PET	Positron emission tomography
PHEO	Pheochromocytoma
PPV	Positive predictive value
SD	Standard deviation
WHO	World Health Organization
